# Rethinking the ethical approach to health information management through narration: pertinence of Ricœur’s ‘little ethics’

**DOI:** 10.1007/s11019-016-9713-6

**Published:** 2016-06-20

**Authors:** Corine Mouton Dorey

**Affiliations:** Institute of Biomedical Ethics and History of Medicine, University of Zurich, Winterthurerstrasse 30, 8006 Zurich, Switzerland

**Keywords:** Health information management, Hermeneutics, Justice, Narrative identity, Self-esteem, Solicitude

## Abstract

The increased complexity of health information management sows the seeds of inequalities between health care stakeholders involved in the production and use of health information. Patients may thus be more vulnerable to use of their data without their consent and breaches in confidentiality. Health care providers can also be the victims of a health information system that they do not fully master. Yet, despite its possible drawbacks, the management of health information is indispensable for advancing science, medical care and public health. Therefore, the central question addressed by this paper is *how to manage health information ethically*? This article argues that Paul Ricœur’s “little ethics”, based on his work on hermeneutics and narrative identity, provides a suitable ethical framework to this end. This ethical theory has the merit of helping to harmonise self-esteem and solicitude amongst patients and healthcare providers, and at the same time provides an ethics of justice in public health. A matrix, derived from Ricœur’s ethics, has been developed as a solution to overcoming possible conflicts between privacy interests and the common good in the management of health information.

## Introduction

Health information management (HIM) is defined as “management of the acquisition, organisation, retrieval, and dissemination of health information” (Medline 2013). Advances in information technology have opened up new possibilities for healthcare information to contribute to clinical care and public health, including links to biomarkers and genetic databases. Parallel to this progress, patients and clinicians hope for health benefits without risk to privacy or intrusive scrutiny. In the healthcare system, information management most often concerns large patient databases. The main ethical challenges pertain to patient informed consent, confidentiality, trust and trustworthiness (Juengst 2014). The development of genomics has widened the knowledge gap between the different stakeholders and increased the complexity of ethical issues regarding the consent process, data sharing, and return of results to donors (Tabor et al. 2011). Challenging conflicts in moral norms have emerged: beneficence versus harm when providing information, respect for persons’ autonomy versus their questionable capacity to assimilate information, and a lack of fairness in the access to support or education for interpretation of genomics information (Appelbaum et al. 2014).

Moreover, technological progress in health information tends to focus attention on the information production tools and the increasing possibilities for data-driven decision-making for health purposes. New healthcare stakeholders from the information technology sciences have entered the medical field with their own health indicators. This issue is recognised by the World Medical Association as an important challenge for medical ethics (WMA 2013). Citizens are also increasingly solicited to contribute directly to health information, to be involved in the decision-making process with their physicians and to benefit from personalised medicine. This last possibility is already widely used to leverage the efficacy of transplantations (Dion-Labrie et al. 2010). In the future, health information systems might ultimately deliver moral recommendations to healthcare stakeholders. Furthermore, the way information is generated and used has an impact on how medical knowledge is shared (Béranger and Ravix 2014). Thus, it is essential that all participants in HIM be involved in the ethical deliberation.

Yet, when patients or healthcare professionals are exposed to multiple HIM situations, they may be subject to conflicting moral recommendations. It is still unclear how to address ethical issues, given the broad spectrum of data covered by health information management. The standard biomedical ethical frameworks are usually targeted to a limited domain: clinical care, research or public health. The management of health information thus requires an accommodating normative ethical basis. It is possible to combine several moral theories in order to cover the entirety of the HIM field, analogous to the model of reflective equilibrium defended by Rawls (1971) and Daniels (1979). But this approach has been criticised, first, on the grounds that it is difficult to access and put into practice (Beauchamp 2004) and, second, because in this model each framework risks losing its distinctiveness and its specific moral justification (Arras 2007, p. 67). Moreover, combining parts of different ethical frameworks to fit the entire scope of health information compromises the coherence of the underlying ethical theory. Therefore, there is need for a more comprehensive overarching model for ethical management of health information.

The aim of this research is to answer the question of *how to manage health information ethically*? In light of the expanding fields covered by HIM, a narrative approach offers an answer to the *how* by providing a complete picture, depicting all characters and their interactions over time. Narration is currently being revisited in its ethical intention, combining principlism, casuistry and virtue ethics, and positioning itself as a hermeneutic enterprise (Brody and Clarck 2014). Ricœur’s work on interpretation is closely related to the narrative aspects of texts, actions and history (1991). Indeed, for the French philosopher, narration is crucial for the mediation between action and moral theory. Based on one’s own narrative identity, everyone is capable of action aimed at the “good” and “obligatory” (Ricœur 1992). Thus, his ethical theory surpasses the binary model of moral rules guided by normative theories, which has been considered too restrictive (Takala 2015).

This paper starts by identifying the narrative dimension of health information management. Narration establishes a bridge between HIM description and interpretation, which in turn leads to Ricœur’s view on narrative identity and ethics. Ricœur’s “little ethics” is then portrayed and applied in a simplified ethical matrix for HIM. It argues that Ricœur’s fundamental aim of *“*the good life with and for others in just institutions*”* provides the appropriate ethical basis for managing health information. The article does not aim to re-interpret Ricœur’s philosophical ethics. Its purpose is to show the relevance of the proposed matrix for the ethical governance of HIM, and to illustrate this with examples in HIM. Finally, possible objections to this Ricœur-inspired approach are briefly addressed.

## A narrative approach to health information management

The production and use of health information should not be reduced to a disembodied collection of data as it engages in a narrative dynamic involving several healthcare stakeholders. Healthcare stakeholders (HCS) refer here to all HIM players, namely patients, donors of biological samples, physicians and other healthcare professionals, but also to information experts, health payers and regulators when they are involved in the management of health information. This section outlines the value of placing the health information elements into a narrative mattering map, taking into account the role of stories.

### The mattering map approach to narration

Montello describes the mattering map as a *how* approach to moral thinking, which helps focus discernment on what matters (2014). In analogy to Montello’s model, the HIM *mattering map* has *voices* that tell the story embedded in HIM, stakeholders as *characters*, the healthcare domain as the *context*, and the purpose and regulations as the *plot*. The subsequent resolution of the HIM story identifies the elements that really matter for patients, their relatives, and observers. Patients (or donors of biological samples) are the voices who tell the story. Other stakeholders can contribute to the tale, but they are not the main narrators. For instance, the physician’s voice can be the one that informs patients and collects data. He also shares patients’ data with other users, and may analyse and interpret the results. Regarding HIM, narration is never about a single voice, and characters reveal their sense of social and self-agency through narrative (Anderson 1997). As in an orchestra, actions and characters are related by correlation and not by causality. Indeed, Montello uses the metaphor of music to explain that the resolution of the plot corresponds to the recovery of “consonance*”*, as opposed to “dissonance*”* (2014, p. S5).

The patient’s voice has long been valued in narrative medicine. As it is not just a matter of subjectivity, objectivity or accuracy, narration reveals something important and authentic about the patient and supports the development of patient’s agency and physician’s understanding (Shapiro 2011). Frank explains the importance of patients’ narrative identity and empowerment for medical decolonisation, i.e. physicians no longer monopolising patients’ stories for their own benefit (1997, pp. 11–14). The value of the narrative is not so much about establishing the absolute truth, but more about emphasising the value of the trust that should govern the patient-physician relationship. This also applies to the relationships between all participants in health information management.

### Examples of mattering maps in HIM

The mattering map helps identify different forms of narration in the field of HIM and reveals ethical dilemmas as *dissonances*, as illustrated in the following examples.

Firstly, some well-known issues compromising the benefit of HIM continue to persist. They notably include the problem of missing voices of patients eligible, but not included in cohorts. Missing voices originate from characters that are more than the mere numerical proportion of non-participants. Larger data set sizes may reduce the influence of misreported data, but cannot make up for what is not included and recorded. Missing voices could regroup those who are not compliant to treatment and could, for instance in tuberculosis or HIV infections, be those who represent a threat of contaminating other citizens or promoting drug resistance. Their identification in the HIM mattering map is not only important to ensure valid data, but also to detect *dissonances* as a failure of the trust agreement between these patients and their physicians, or a lack of transparency and trustworthiness between HIM characters, or a deception in the construction of the common good because of biased scientific publications.

Secondly, the mattering map can reveal dissonant HIM due to the secondary use of data, i.e. when patient/donor information is provided to third parties in the absence of patient/donor consent, or even without physicians’ knowledge. Potential breaches in confidentiality and mistrust in the management of health information require better guidelines governing access to health databases. This is especially pertinent with biobanks. Initially based on human material archived for clinical or research purposes, biobanks have evolved towards large-scale genetic research, including the joint analysis of phenotypic and clinical data from patient cohorts (Wain et al. 2015). Voices and characters are better identified with the addition of data on phenotypes and medical contexts. This combination supports innovative research and more personalised diagnosis and treatment for patients. Thus, new HIM mattering maps are emerging in genomics. For instance, the return of incidental findings to donors follows various plots, depending on the findings and the type of informed consent. Considering the family members of the donor, Lenk and Frommeld have analysed different models, in which, through the description of actors, roles and contexts, the narrative components are revealed (2015).

Thirdly, in the context of personalised medicine, the HIM mattering map has to consider the emergence of predictive components of increasing precision, which contribute to more specific diagnostic tests, refined disease classification and individualised treatments (Jameson and Longo 2015). As a result, HIM faces an ethical dilemma between an abstract promise of truth carried in the emergent information, and a concrete decision to be reached on the communication and use of this information. The patient’s voice might fade behind the intrusion of these new medical scientific findings or, quite the reverse, enforce the patient’s participation in the medical decision. Each individual could be the heroine/hero in her/his own health story. This is already noticeable in medical screening, with the publication of amazing survival stories following early cancer detection. There are also negative stories of overdiagnosis that challenge the validity of screening advantages (Moynihan et al. 2014). These conflicting stories around the issue of screening exemplify another risk of narrative dissonance in HIM, e.g. the accuracy of the decision when there is insufficient evidence of the predictive value of new biomarkers.

Fourthly, dataset linkages, multiple uses of data in large medical databases, as well as the increasing availability of individual health data from the internet or mobile devices, have contributed to the development of big data in health. Big data represents the process that uses and reuses health and research data with the help of sophisticated digital processing and algorithms; the objective is to identify new health patterns based on data mining methods, which open new perspectives for future medical research and are thus different from traditional hypothesis testing methods (Nuffield Council 2015, pp. 15–16). The mattering map recognises these patterns as new types of plots in HIM, disconnected from inceptive single voices and challenging the roles and responsibilities of traditional characters. Indeed, data mining can be performed by information experts without the intervention of physicians or the need for close relationships with patients/donors. The application of big data algorithms is dramatically increasing complexity in HIM narration. On the one hand, big data supports the detection of unexpected or rare patterns that hypothesis-testing research ignores. On the other hand, the correlation of findings with disease or prognosis may be unclear. This evolving but still uncertain medical context requires revisiting ethical views on individual consent, privacy, public health interests, information property, altruism and commercial development (Vayena et al. 2012).

Finally, advances in data size and analytic methods in HIM could be seen as a resurgence of scientific positivism, adding new narrative paths in the research for truth, and challenging the moral references for medical and bioethical judgement. Depending on the queries, numerous different plots could be revealed, which not only enhance scientific research, but also carry new patterns in moral thinking for HIM itself.

These HIM examples are not stand-alone and can be combined into a wider mattering map. The plurality of sources and connections constantly enlarges the HIM domain. Nevertheless, big is not always better for the management of health information (Toh and Platt 2013). Therefore, a narrative approach would mitigate the eagerness of data-driven medical judgement, and stimulate reflection in order to better *interpret*, i.e. discern and understand the type of precision, truth, and voices that matter in the management of heath information.

## The passage by interpretation and the link with Ricœur’s philosophy

The narrative approach opens up different perspectives of interpretation permitting an understanding of the patient, the message embedded in the health information and the behaviour of those using the information. *The model of text interpretation can be applied to HIM* following the quantitative (data processing) and qualitative (information management) aspects. This duality mimics the distinction between the locutionary part (the sentences) and the illocutionary part (how sentences are expressed) of a text, both of which meanings need to be analysed (Ricœur 1973). The theory of text and the theory of action have been developed separately in philosophy, and both theories can lead to a dichotomy between an explanation of structure and an understanding of motivation. Ricœur refutes this dichotomy because there is a continuous interference of human action in the course of events and vice versa, and this holds for text and action as well (1991). As a result, everything that can be understood from human action and history can be interpreted as a text.

This hermeneutical approach to action as a text is relevant for both historical and fictional narrative. At the intersection of these two key classes of narrative there stands human identity, which has to be understood as a *narrative identity*. Ricœur calls narrative identity the assignment of a specific identity to an individual (or a community) who is the subject of an action and who tells the story (1988). He differentiates two poles in the narrative identity: sameness and selfhood (1992, pp. 115–125). *Sameness* is about the question “what I am”, the usual identity conception of being the same person, different compared to others and permanent throughout life and its course of events. Sameness concerns natural traits, physical, biological, and genetic characteristics. *Selfhood* is about “who I am”, the very specific self, who is reflecting, non-permanent, adaptable, and capable of determining its own life. Selfhood implies an intimate relationship to otherness, and comprises the idea of faithfulness to the self “en devenir” (i.e. in the process of developing). Selfhood has an ethical dimension that evokes the agency freedom advocated by Sen (1985). Furthermore, the so-defined narrative identity can be applied to the individual as well as to the community, and these identities can be combined to build a common story.

The management of health information finds an echo in Ricœur’s account of narrative identity. The construction of the plot brings to life the actions of the characters. Ricœur considers that this transposition from actions to characters establishes the characters’ narrative identities in their two dimensions of sameness and selfhood (1992, pp. 140–143). Indeed, when patients/donors provide data, they share their narrative identity as sameness, adding their voice to the other same. Their narrative identity as selfhood makes them capable of deciding whether or not to participate, to give up rights to some personal sameness, to interact with the other stakeholders and institutions in a responsible way, to receive feedback information and to adapt accordingly. Each patient is also part of a community and contributes to the common narrative identity. Interpretation of the narrative HIM supports both the self-comprehension of a given participant and the comprehension of others in the medical and social community. Consequently, private and community goods are intimately interconnected, and it is possible to overcome the classical ethical dilemma between privacy rights and common good.

Furthermore, the interpretation of narrative HIM falls within the dimension of temporality (Ricœur 1984, pp. 52–87). The plot includes a succession of events with the possibility of unexpected events or patterns, depicting the *narrative time*. The concept of narrative time is particularly important with new types of HIM since biomarkers or population patterns can be delivered at a time when their value and possible medical use are not yet understood. As it combines individual and community aspects, as well as temporality, Ricœur’s concept of narrative identity supports a more comprehensive model for interpreting HIM narration, compared to the relatively narrow model of narrative medicine for specified clinical settings as developed by Charon (2001). This enriched model helps progress from interpretation to comprehension of HIM and establishes a first ethical perspective.

Ricœur further defends the passage from narrative identity to ethics. He identifies different roles for characters, with the possibility to be both subject and actor in a story. Thus, the patient is a human being acting and suffering, and this attests the correlation between narrative and ethics (1992, pp. 145–164). In the narrative HIM, actions are evaluated: patients and healthcare providers are actors, who can be approved or admonished, and their individual narrative identity is exposed to the regard of others. The narrative identity transforms a passive character into an active one, capable of deciding and acting accordingly. Specifically, it confers self-determination and accountability to the role. Therefore the narrative theory serves as mediation between the theory of action and the theory of ethics. Moreover, Ricœur’s ethical theory develops the promise of sharing between the two narrative identities, personal and collective. Such a promise of sharing is essential for the ethical governance of HIM. This paper will thus further explore the ethical vision proposed by Ricœur.

## Overview of Ricœur’s “little ethics”

Ricœur first proposed his “little ethics” in the book “Oneself as Another” (1992). He then completed his ethical work in further lectures and in two books on “The Just” (2000, 2007). He differentiated between the terms *ethics* and *morality*. Ethics is about what is considered to be good (teleological, Aristotelian perspective), and morality is about what imposes itself as obligatory (deontological, Kantian perspective). He proposes a new architecture for ethics which explores *“*the capacities and incapacities that make a human being a capable, acting and suffering, being*”* (2007, p. 2). The concept of agency conveys this capability to act and to be accountable for one’s own actions. Agency expresses itself through the narrative individual and collective identities (2000, p. 3). Thus, ethics is not about the identity of things, data or a disembodied healthcare information system, but about *moral agents* (in our topic, healthcare stakeholders). This analysis supports the idea that the right metaphor for the healthcare information system is not a business or warehouse model, but rather a human organisation. Indeed, Ricœur’s work is about participative and communicative human organisation, with the concept of “*The Just*” influencing all human actions.

Ricœur’s ethical philosophy rests upon the following three propositions: “(1) the primacy of ethics over morality, (2) the necessity for the ethical aim to pass through the sieve of the norm, and (3) the legitimacy of recourse by the norm to the aim whenever the norm leads to impasses in practice” (1992, p. 170). This article describes the three steps consecutively.

### The primacy of ethics over morality

Ricœur names “anterior ethics” the ethical aim of the “good life, with and for others, in just institutions”. Within this anterior ethics, he distinguishes three ethical values that are linked, but do not overlap: self-worth, reciprocal trust, and participative justice.

#### Self-worth

The teleological philosophy of *the good life* (sense of life in its entirety, not only biologic or fragmented) includes the notion of good virtuous actions, such as standards of excellence for physicians, as well as the good life towards which all these actions are directed. When they interpret their actions, the agents develop a self-interpretation which becomes self-esteem at the ethical level (1992, pp. 172–179). Self-esteem corresponds to the good applied to actions.

#### Reciprocal trust

Solicitude, as described by the good *with and for others* is the ethical phase about reciprocity, sharing and living together. Solicitude is based on the exchange between giving and receiving. Although this exchange in a friendship relationship is hypothetically equal, most often a dissymmetry appears because the initiative for the exchange comes either from the self or from the other. Based on an ethical response of benevolent spontaneity (e.g. the patient) or spontaneous compassion (e.g. the clinician), solicitude aims to establish equality in dissymmetrical conditions, the self becomes another among others. This element of similitude implies trust and belief in one’s own worthiness.

#### Participative justice

The sense of justice is the third phase of this anterior ethics. When a relationship encompasses many citizens from a community or nation, the notion of life concerns the institutions. Institutions are defined by the structure of living together bound by common customs and not by constraining rules. The ethical aim introduces the dimension of justice as proportional equality for each. Ricœur identifies two faces of the just, one teleological towards the good and one legal towards the judicial system and the law of constraints. His anterior ethics focuses on the teleological face and concerns the sense of justice, which combines both aspects of sharing: “being part of” and “receiving a share of”. This dual view precludes opposition between the individual and the society. The unjust is synonymous with unequal, taking too many of the advantages or not enough of the burdens. This sense of justice extends equality to the entire humanity.

### The normative or deontological level

The second proposition analyses the moral level (i.e. the norms), which corresponds at the ethical level to self-esteem, solicitude and sense of justice. The formalism of the norms represents the obligations, which ensure a just distance between HCS in all plots. Ricœur believes that the passage through the norm enriches the anterior ethics (1992, p. 203). Autonomy, respect for others and legitimacy of distributive justice are the dominant deontological values.

#### Autonomy

At the deontological level, self-respect corresponds to the ethical aim of self-esteem. Ricœur refers to deontological Kantian morality and the corresponding principle of autonomy because the same subject has both powers of giving orders, and of obeying or disobeying. Maxims are submitted to the rule of universalisation and associated with the idea of duty. Self-esteem, which does not pass the test of universalisation, is “self-love”, a penchant for evil that affects the freedom to act and the capacity for being autonomous.

#### Respect for others

Solicitude corresponds at the moral level to respect for others and to the second Kantian imperative of persons as an end in themselves. There is a need to (re)establish reciprocity in front of the initial dissymmetry between agents and subjects, due to the exercise of power of one will over another will. The answer of moral norms is a “no”, a prohibition of all the forms of evil, violence, and humiliation, whereas, at the ethical level, solicitude was affirmative in compensating the dissymmetry in self-esteems.

#### Legitimacy of distributive justice

Finally, at the deontological level, Ricœur considers a strictly procedural justice, as developed by John Rawls in opposition to Utilitarianism (1971). The legal face of the just is separated from the good, and rests upon the tradition of the social contract, a founding fiction that is anti-teleological. Ricœur, however, challenges the procedural justice of Rawls since the justification of equality and inequalities has no recourse to anterior ethics. For him, the fiction of the social contract is compensation for the forgotten ethical foundation of *“*the desire to live well with and for others in just institutions*”* (1992, p. 239).

### “Posterior” or applied ethics

Applied ethics follows the third proposition and represents the other face of ethics, i.e. wise recourse to the ethical aim when norms face conflict in practical situations. Ricœur develops practical wisdom in order to deliberate justly at the three previous levels of the institutional environment, the plurality of persons and the universal self (1992, p. 240). Sharing in practice highlights the recourse to values of equity, confidentiality and the ability to judge wisely.

#### Institutional environment and equity

The rule of justice includes an element of ambiguity because of the diversity of the primary goods to be distributed. The fairest rules of justice face the issue of arbitrage between different goods that delimit different spheres of justice. The indeterminacy in political power may open the door to domination, totalitarianism and exploitation. Following Aristotle, Ricœur appeals to equity as practical wisdom in order to correct possible conflicts in the application of the rules of justice.

#### Plurality of persons and confidentiality

With regard to respect for others and the second Kantian imperative, conflicts can arise in the application of the universal law and the need to arbitrate between the multiple duties that pass the test of universalisation. The dissymmetry in interpersonal relations has the potential for conflicts, with a risk of arbitrariness when the idea of protection replaces the idea of respect. This distinction is complex in novel situations, such as biomedicine, where progress and technology also include an imperative of responsibility towards the future generation. Ricœur appeals to a “critical” solicitude as the form of practical wisdom in the situation of conflicting interpersonal duties.

#### Universal self and the ability to judge wisely

Finally, the principle of autonomy as self-legislation is subject to moral conflicts in situations in which moral judgement has to arbitrate between universal rules of morality and contextual moral values. Ricœur opts for a critical argumentative ethics and refers to Rawlsian reflective equilibrium. In posterior applied ethics, practical wisdom implies a real discussion with mutual recognition and openness to truth or to meanings that are foreign to the self. It is *recognition* that structures the ethics from self-esteem to solicitude and to justice, i.e. applied ethics is developing backwards from the idea of justice to respect for others and finally respect for *oneself as another* (1992, pp. 273–274, 280–281).

## A simplified matrix for health information management based on Ricœur’s ethics

Ricœur applied his “little ethics” to the medical domain by developing three levels of moral judgement: “prudential” with practical wisdom in posterior applied ethics, “deontological” at the normative level, and “reflexive” at the level of anterior ethics (Ricœur 2007, pp. 198–212). In the situation of medical practice, Ricœur’s starting point for consideration is posterior ethics. He regards the relationship between suffering patients and physicians as the basis for ethical significance in bioethics (Ricœur 2007, p. 198). Ricœur further explores the dimensions of prudential judgement by comparison with judicial judgement, which involves a greater number of protagonists (2007, pp. 213–222). He recognises that the concrete act of medical decision-making involves a growing number of protagonists coming from the medical sciences or public health. New issues are raised such as “colonisation” of the medical act by rapidly advancing biologic and genetic knowledge (p. 215), or “fairness” in relation to medical costs at a population level (pp. 216–217).

This paper has used a similar approach to build an ethical matrix in the field of health information management (Table 1). The proposed ethical matrix includes the three levels of judgement from Ricœur’s “little ethics” as columns: *anterior ethics* (or reflexive), *moral norms* (or deontological) and *posterior applied ethics* (or prudential). The design of the matrix integrates the second dimension of the ethical aim of the “good life, with and for others, in just institutions” aligned with the three steps of ethical considerations for stakeholders: *self, others,* and *society*.

**Table 1:** Ethical matrix for health information management (HIM), derived from Paul Ricœur’s work (1992, 2000, 2007)

HIM healthcare stakeholders (HCS)	Anterior ethics (teleological, Aristotle)	Moral norms (deontological, Kant)	Posterior, applied ethics (to HIM)
Self	Aim at the good life: *Good as actions:* Virtues Deliberation on means to reach them Vocation Standards of excellence *Good as self-esteem:* Narrative unity of life, narrative identity Self-esteem different from esteem of myself	Self-respect: Goodwill Universalisation of the moral law Autonomy as moral selfhood, practical reason, free choice *Issues*: Passivity within autonomy Acts not imputable to agent Perversion: self-love Misuse of free choice, inclination towards evil	Patient–physician pact based on: Trust agreement (on each side) Agency empowerment Levelling out dissymmetry of knowledge and respect Building alliance to decide and act *Fragility of the pact:* Patient preferring dependency Clinician humiliating patient
Others	For and with others: *Solicitude (concern for others):* As a vital extension of self-esteem Friendship and reciprocity Equal good to others as another self Sharing Living together Benevolent spontaneity *Opposed to suffering*	Respect for others: Golden rule Norm of reciprocity, benevolence Dissymmetry between active and passive roles *Issues:* Torture, violence, humiliation as destruction of others’ self-respect Exploitation Betrayal	Medical contract (mandate): Confidentiality, professional secrecy Sharing truth, collective narrative Patient information and consent Professional codes, patient rights Trustworthiness between HCS *Fragility of the contract:* Boundary between clinical care and research
Society	In just institutions: *Living together:* Participation in the life of institutions Common mores No split between governing and governed Plurality: anonymous but irreplaceable Action in concert but some are forgotten *Justice:* Justice as virtue, desire for just and good actions and things Equality: distributing as sharing and repairing	Principles of Justice: *Public health laws:* Legal authority Legitimation *Justice:* Equality: distributive justice Social contract *Issues:* Arbitrage between legal authority and common good morality Ignorance of teleological foundation	Common good partnership: Public healthcare agency Communication, dissemination, transparency Solidarity, equity, access to care Concerns for missing/neglected patients *Fragility of common good partnership:* No obligation to be healthy No obligation to cure Unreasonable expectations Lack or excess of prudence Hurdles to sharing

The matrix connects HIM with Ricœur’s ethics using a *Ricœurian path* of reflection. Ricœur supports a reflective process in medical judgement, for instance when he questions the link between “the request for health and the wish to live well” (2007, p. 212). An analogous parallel for HIM would question the evidence of a positive relationship between profuse health information and improved well-being. For descriptive purposes, this paper progresses through the matrix line by line. As in Ricœur’s medical judgement, the HIM context of the patient-physician interaction is used as a basis for reflection, which can then be extrapolated to alternative HIM situations.

### Self

The self-esteem developed with the aim of “the good life” expresses itself as the moral norm of self-respect and autonomy. Patients choose freely to participate in HIM and give up rights to their personal data. However, the practical situation is dissymmetrical, the physician having more knowledge, information and position power than the patient. Patient agency needs to be empowered, and the patient-physician alliance will develop agency, providing that there is trust on both sides. This means, in particular, that the patient trusts and follows the physician’s advice, and that the physician trusts the patient’s voice and tries to fill the gap of the patient’s ignorance. Physicians are also increasingly accepting scrutiny of their personal work by the other participants involved in the management of health information.

In the posterior applied ethics column, HIM relies on the patient-physician pact based on trust, similar to other medical situations based on “agreement regarding trust” (Ricœur 2007, pp. 199–200). In the absence of the corresponding moral norms of self-respect and autonomy (deontological column), this trust pact is weakened, with the practical risk that patient participation in decisions regarding HIM would be neglected, and as a result the patient would feel humiliated or unable to overcome passivity. Thus, suffering patients are vulnerable to the physicians’ abuse of power or failure to fulfil their expectations regarding management of their personal health information. In the anterior ethics column, the patient is considered as indivisible regarding clinical, biological, psychological, and social identities. This narrative unity stresses in turn the importance of patient agency and the roles of physicians who face the singularity of each patient in practice. The physicians’ appropriate training and experience should help to overcome the dissymmetry of knowledge between them and the patients, as well as other HIM agents. More generally, health providers should be accountable for empowering patient agency and, as a result, the trust agreement would be maintained for ethical management of health information.

### Others

The anterior ethical aim of solicitude as “a good life with and for others” expresses itself as the moral obligations of respect for others and benevolence, which in turn support the posterior applied values of confidentiality, patient autonomy, applications of the professional code and respect for patient rights. Practical wisdom encourages a collective narrative in the management of health information including trustworthiness between all healthcare stakeholders. The norms of reciprocity and benevolence protect those who are passive and vulnerable because of lower capacities, and justify equal consideration of others as another self.

The fragility of this medical contract comes from the difficulty of differentiating between the HIM for clinical care and the HIM for healthcare research, with research requiring more stringent norms. Ricœur identifies this issue when he points out that “the human body is both a personal being and the observable object of scientific investigation as a part of nature” (2007, p. 206). Therefore, individuals can be observed as an object, and the corresponding measurements can be used independently of the donor, with the risk of misuse of health information and harmful exploitation. Following Ricœur’s approach to solicitude and benevolence for and with others, practical wisdom for judgement in HIM should follow “the three rules of medical secrecy, the patient’s right to the truth, and informed consent” (2007, p. 211).

### Society

Solicitude is necessary for sharing data between HCS, but not always sufficient. A society with just institutions will provide equitable access to and use of health information results, as this participation is the foundation of a common morality. The theme of justice is represented in the first column of the matrix and culminates in the line “society” with the establishment of a just distance in the relationship with all other human beings. The concept of justice in society then evolves horizontally across the three columns from sense of justice, to social justice based on legislation and procedure, to justice as equity in the face of practical problems.

A broader view than the patient–physician pact and the three prudential rules in medical judgement is required for HIM at the society level. More precise biomedical information from scientific experts changes the paradigm of medical decision-making towards a more technical approach (Ricœur 2007, pp. 214–215). Furthermore, population statistics and economics shift the decision-making process from the suffering individual to the protection and sustainability of public health according to norms of distributive justice (pp. 216–217). Justice is thus relevant at the three levels of judgement, i.e. all HCS have to be part of the governance of HIM and organise their co-existence; the legal norms should protect patient privacy and legitimate public health actions; applied ethics should ensure equity with a just sharing of burdens and a just dissemination and interpretation of health information.

## Application of the ethical matrix reflection path to HIM examples

The examples described with mattering maps in “A narrative approach to health information management” section are analysed using the ethical matrix derived from Ricœur’s ethics. The points of weakness that were identified in the Introduction (first paragraph) serve to identify the *ethical issues* to be reflected upon. The examples are discussed as separate cases for didactic reasons.

### Trust and trustworthiness: missing patients and validity of HIM

In clinical practice, a bottom-up participation to common good would minimise the number of missing patients in research databases (*society*). Communication, trust and trustworthiness between all HCS would be encouraged, including the possibility of combining social support in the community network (*others*). The steering committees for HIM should consider field knowledge and include representatives of first-line data-collectors, patients and social communities. Drafts of publications should be shared and discussed prior to publication.

### Informed consent, respect for persons’ autonomy versus their questionable capacity to assimilate information: HIM and secondary uses of data

Concerning access to health information by third parties, applying the trust agreement and the medical contract as described in the matrix would solve the ethical issue of patients suffering and feeling betrayed following inappropriate use of their data and consequent infringement of their privacy. In practice, first-line physicians ought to be informed by third parties about all aspects and possible future developments of healthcare information to which they are contributing (*others*). While building trust with patients in the iterative process of consultations, the physician should also help patients reach an adequate level of comprehension and provide appropriate information concerning the current and future management of their healthcare data. As their level of HIM literacy improves, patients can aim for a status of associate, sharing decisions on the management of their health data and information with healthcare professionals (*self*).

### Consent process, data sharing: issue of broad consent

As for biobanks, some hospitals have introduced broad consent covering the future use of patients’ coded or de-identified health information. This means that patients are not fully informed at the time of consent since nobody can know all the future uses. The consent might be legally right, depending on the specific country of legislation context. However, Ricœur’s ethical architecture supports the primacy of ethics over legislation, meaning that just institutions are not only institutions ruled by law, but are participative institutions, with shared values for just and good actions. In practical situations of conflicting judgements on consent process and data sharing, the matrix refers to the anterior ethics arbitrage based on solicitude and the sense of justice as sharing and participation. Therefore, a broad consent should require ethical reflection involving all HCS before its possible acceptation. The World Medical Association has also advocated the primacy of ethics over law and does not favour unconditional broad consent (WMA 2003, 2015).

### Beneficence versus harm when providing information: personalised healthcare

In the genomics example on the return of incidental findings to patients or their family, no model for information and consent has been considered as ethically optimal. Appelbaum et al. have recommended a better education for donors. They also consider researchers to be accountable for providing this service and reducing the potential harms related to health genomics information (2014).

New medical sciences applied to personalised medicine are provoking the emergence of new biomedical patterns of sameness, disrupting a patient’s/donor’s self-interpretation. Narrative identity as selfhood needs to clarify one’s self-understanding continuously. Furthermore, incidental findings in genomics, or screening results for early disease detection, confirm that the narrative time is important: new findings could emerge unexpectedly in the patient/donor–physician/researcher relationship.

In the matrix, the applied ethics column supports Appelbaum’s proposition, i.e. the empowerment of donor/patient agency, as well as the researchers’/physicians’ responsibility for levelling out the dissymmetry of knowledge regarding genetic testing (*self*). As a result, shared decision-making is possible. The matrix challenges the usual ethical approach, in which researchers or physicians are left alone to obtain meaningful informed consent from donors or patients. The teleological aim combining *justice* and *living together* drives the legitimacy of the moral decisions for HIM in genetic testing and medical screening, and helps to match the research tempo with the common good (*society*). Moving upwards in the anterior ethics column, the sense of justice will lead to benevolent sharing with others and protecting the self-esteem of the most vulnerable. This ethical deliberation results in recognition of the specific narrative identity of each participant, who thus becomes capable of acting and deciding on genomics testing or medical screening. Furthermore, based on Ricœur’s “circular” concept of narrative identity and temporality (1988, pp. 241–249), the matrix proceeds in a *Ricœurian path* of reflection and puts critical argumentative ethics on a long-term footing. This permits a settlement between the two different times of abstract findings and concrete decision-making. In practice, the ethical matrix supports the disclosure of incidental findings under conditions of benevolent reciprocity and time. It thus opens a reflection path for human governance of data-driven health management in personalised medicine.

### Fairness in HIM: objectification of individuals versus new scientific advances with big data

The primacy of ethics over legislation holds for the management of health information with big data. Big data escapes the traditional narrative of medical practice, clinical research and the patient/donor–physician/researcher relationships. The “three rules of medical secrecy, the patient’s right to the truth, and informed consent”, as well as the concept of individual indivisibility are challenged. Moreover, the high speed of big data development requires continuous normative adaptation to legitimise their use.

In the matrix, the ethical reflection path for big data relies on the concept of justice developed in the three columns of applied posterior ethics, moral norms and anterior ethics. The sense of justice is the key element as it supports sharing, i.e. making available the sources, algorithms and results of big data, as well as repairing when findings have harmed people. The corresponding principles of social justice and the equitable dissemination of knowledge will favour a bottom-up “democratic” participation around the governance of big data. Therefore, citizen education and participation would protect patients and health providers from uncontrolled fears leading to an unreasonable principle of precaution, as well as from potential hidden coercion of public health or absence of prudence in the use or commercialization of big data. Such a democratic management of health big data could enhance the ethical reflection of HCS, increasing their self-esteem and agency, and reduce the risk of medical or public arbitrariness.

Finally, this paper has mentioned, at the end of “A narrative approach to health information management” section, the possibility that moral queries in big data could unveil innovative normative patterns in moral thinking for health information management. This could challenge the current normative principles of justice. The matrix helps to analyse the issue since procedural justice does not pre-empt the ethical construct for HIM. The process of deliberation starts at the level of posterior ethics with openness and prudence in the founding of common good. Then, normative development proceeds by adjustment using anterior ethics, gradually revising public health legitimacy and distributive justice. Moreover, the matrix sets limits in the face of a possibly misleading moral guidance of big data, with the anterior ethics column establishing a clear ethical aim. For instance, the matrix differentiates between esteem of myself (self-love) and self-esteem, and would limit excessive health demands.

#### In summary

The matrix derived from Ricœur’s “little ethics” is an appropriate ethical framework for application to the management of health information because it emphasises patient agency, trust agreement between HCS, and justice as equal and equitable participation to the common good. Ricœur’s ethics takes into account the contributions of other ethical approaches, such as the Kantian and Rawlsian theories, but ensures the primacy of anterior ethics over moral and legal norms. The recourse to anterior teleological ethics allows the possible moral conflicts to be overcome and leads to wise and shared decision-making in the management of health information in medical practice, research and public health. This model of continuous ethical reflection could be transferable to other technology-transformed healthcare activities.

## Brief critical appraisal of Ricœur’s ethical approach

As a result of the widely held view that he was a philosopher of great complexity, Ricœur is rarely referred to in biomedical ethics (Potvin 2010). His extensive work is built up architecturally in successive books comprising a continuous in-depth reflection which looks for coherence between ancient and recent philosophical theories. Therefore, there is a risk of favouring only part of his work, or of disregarding it as a whole. In this paper, the focus has been placed on Ricœur’s “little ethics”, and some objections to this ethical approach need to be briefly addressed.

First, reference to the good life might convey the impression that Ricœur’s ethics is simply about virtues and care ethics. Care takes into consideration patients’ voices and desires, but usually conflicts with a depersonalised public health orientation. Although his approach has some points in common with care ethics, Ricœur does not reduce justice to friendship and equal consideration for others (Van Stichel 2014). He justifies the teleological aspect of justice by a passage through the norms of distributive justice and the political social contract. His ethical approach favours the concept of common good, rejection of injustice, and solicitude/love within the philosophical domain.

Second, the possible reproach of the is/ought fallacy could be raised. The narrative approach of HIM can be considered as a descriptive one (“is”) and therefore as not having to lead directly to prescription and moral norms (“ought”). Ricœur’s ethics avoids any direct connection between description and prescription when he introduces the passage by interpretation (1992, pp. 169–170). This approach is supported by his philosophical work on hermeneutics. Furthermore, there is no such thing as a pure “is”, and empirical data are not only facts, but also include experiences, cultural and normative elements (Dunn and Ives 2009). This holds for HIM, with normative influences having an impact on HIM design and purpose.

Third, the choice of a teleological philosophy as anterior ethics may be challenged. Anterior ethics is “de facto” teleological. It is difficult to find alternatives. Ricœur indicates that Mill, but also Kant referred to some teleological goodwill, albeit in a soft way. Recent ethical frameworks for healthcare have introduced economic wellbeing as an ethical theory basis, justified by the need to have sustainable healthcare (Faden et al. 2013). The value is then the sustainability of something considered valuable, and refers to teleological equality as sharing with those in the future. In this example, economic wellbeing cannot pass the test of anterior ethics directly. It belongs to the moral norms.

A fourth objection could be that this overarching ethical framework is too complex and theoretical. Yet, far from being too theoretical, this ethical oversight can already be detected in the management of health information in practical fields, such as rare diseases, where patient agency, patient information and consent, physician accountability and the distinction between care and research are extensively developed (Duchange et al. 2014). Furthermore, this paper has demonstrated that the ethical matrix adapted from Ricœur’s “little ethics” supports a deliberation process that can address practical issues in emergent narratives of HIM, such as those raised by personalised healthcare.

## Conclusion

The ethical management of health information concerns all healthcare stakeholders, healthcare professionals, as well as patients. This paper has suggested that a narrative approach to HIM is able to connect individual and collective narrative identities. Moreover, this narrative approach has similarities with Ricœur’s dual concept of narrative identity as sameness and selfhood. Using interpretation as mediation between narration and prescription, Ricœur shows the importance of moral agency, and that the capacities of acting and suffering belong to an ethical order. Ricœur’s “little ethics” inspires a useful ethical framework for the management of health information, helping to prevent the tendency to reduce patients to mere data, and healthcare providers to mere data gatherers, in addition to contributing to solving moral conflicts in the healthcare information context. The ethical matrix proposed in this paper combines the dimensions of self, others and society with the dimensions of anterior ethics, moral norms and applied ethics. The dominant values of agency, trust and justice help to guide practical wisdom in managing health information.
